# Effect of Bromfenac on Reducing Neuroinflammation in an Ischemia–Reperfusion Glaucoma Model

**DOI:** 10.3390/cells13121046

**Published:** 2024-06-17

**Authors:** Si-Eun Oh, Jie-Hyun Kim, Chan-Kee Park, Hae-Young Lopilly Park

**Affiliations:** 1 Department of Ophthalmology, Bucheon St. Mary’s Hospital, College of Medicine, The Catholic University of Korea, Seoul 06591, Republic of Korea; 2 Department of Ophthalmology, Seoul St. Mary’s Hospital, College of Medicine, The Catholic University of Korea, Seoul 06591, Republic of Korea

**Keywords:** glaucoma, neuroinflammation, retinal ganglion cells

## Abstract

In the context of glaucoma, intraocular pressure (IOP) and age are recognized as the primary factors contributing to its onset and progression. However, significant reductions in IOP fail to completely halt its advancement. An emerging body of literature highlights the role of neuroinflammation in glaucoma. This study aimed to explore Bromfenac’s anti-inflammatory properties in mitigating neuroinflammation associated with glaucoma using an ischemia–reperfusion (IR) glaucoma model. Bromfenac’s impact on microglia and astrocytes under pressure was assessed via Western blotting and an enzyme-linked immunosorbent assay. Immunohistochemical staining was used to evaluate glial activation and changes in inflammatory marker expression in the IR model. Bromfenac led to the downregulation of inflammatory markers, which were elevated in the conditions of elevated pressure, and necroptosis markers were downregulated in astrocytes. In the IR model, elevated levels of GFAP and Iba-1 indicated glial activation. Following Bromfenac administration, levels of iNOS, COX-2, and PGE2-R were reduced, suggesting a decrease in neuroinflammation. Furthermore, Bromfenac administration in the IR model resulted in the improved survival of retinal ganglion cells (RGCs) and preservation of retinal function, as demonstrated by immunohistochemical staining and electroretinography. In summary, Bromfenac proved effective in diminishing neuroinflammation and resulted in enhanced RGC survival.

## 1. Introduction

Glaucoma is a progressive optic neuropathy characterized by the progressive loss of the retinal ganglion cells (RGCs) [1]. Intraocular pressure (IOP) and age are considered the most important attributable factors in the onset and progression of glaucoma [2]; nevertheless, sufficient lowering of IOP does not halt the progression of glaucoma completely. Neuroinflammation is another critical factor that is generally accepted as a key player in the pathology of progressive optic neuropathy in glaucoma. There is an increasing amount of literature on neuroinflammation in glaucoma using various animal models and the human glaucomatous optic nerve head (ONH) [2,3,4,5,6,7,8,9,10].

Resident astrocytes and microglial cells are responsible for the primary inflammatory responses in the central nervous system (CNS) [2], and astrogliosis and microgliosis have been observed in the neuroinflammation of glaucoma [3]. In our earlier investigation [11], we observed neuroinflammation characterized by the activation of glial cells in a systemic hypotensive model that mimics blood flow instability. This inflammation led to scleral fibrosis and played a role in advancing glaucoma by contributing to RGC death. We observed neuroprotection through the inhibition of pathways associated with neuroinflammation [12]. However, the most critical clinical impact of neuroinflammation occurs during acute increases in IOP, and in considering the therapeutic options applicable in such cases, we deliberated on the potential of Bromfenac.

As all of the approved treatments for glaucoma target IOP lowering [7], there has been a growing demand for alternative treatments targeting neuroinflammation. As neuroinflammation may act as a double-edged sword, having both harmful and supportive effects on RGC survival, new therapeutic approaches should not just inhibit the inflammatory process but also work as immunomodulatory agents [2]. Bromfenac is a nonsteroidal anti-inflammatory drug (NSAID) marketed as an ophthalmic solution and is widely used for the treatment of postoperative ocular inflammation following cataract surgery [13]. Its main mechanism of action is to block cyclooxygenase (COX), inhibiting the synthesis of prostaglandin (PG) to reduce inflammation, and Bromfenac is a relatively COX-2-selective agent [14]. Bromfenac demonstrates sufficient ocular penetration upon topical administration. Previous animal studies have demonstrated that Bromfenac ophthalmic solution rapidly reaches measurable levels in all major ocular tissues, including the retina. Peak concentration is observed at 2 h, with detectable levels maintained for over 24 h [15,16].

The ischemia–reperfusion (IR) injury animal model is a glaucoma animal model used to observe neuroinflammation, and reactive microglia have been identified in the IR model, similar to what is observed with optic nerve axotomy, ocular hypertension, and human glaucoma [17]. We used an IR injury animal model to determine the anti-inflammatory effect of Bromfenac on neuroinflammation in glaucoma, and furthermore, we investigated whether the suppression of neuroinflammation resulted in neuroprotection. Initially, we examined the impact of Bromfenac on microglia and astrocytes under elevated pressure. Next, we assessed glial activation and alterations in inflammatory marker expression in the IR model. Lastly, we investigated the effects of Bromfenac on RGC survival and electrophysiological function in the IR model.

## 2. Materials and Methods

### 2.1. Cell Culture in Pressure Chamber and Bromfenac Application

The BV-2 microglial cell line from Elabscience (Catalog No. CL-0493, Houston, TX, USA) and the DI TNC1 astrocyte cell line from ATCC (Catalog No. CRL 2005, Seoul, Republic of Korea) were cultured in DMEM (Gibco, Grand Island, NY, USA) supplemented with 10% fetal bovine serum (Gibco) and 1% penicillin/streptomycin at 37 °C in a humidified atmosphere with 5% CO_2_. Cells were passaged when they were approximately 80% confluent. Depending on the experimental conditions, the cells were inoculated into culture dishes. Once the cells adhered to the surface, subsequent studies were conducted. After exchanging the culture media and applying 0.1% Bromfenac sodium hydrate ophthalmic solution (Bronuck^®^; Taejun Pharm. Co., Ltd., Seoul, Republic of Korea) to achieve a stock concentration of 8 mM, BV-2 microglial cells and DI TNC1 astrocytes were pretreated with 24 h of starvation. The cells were then placed in a pressure chamber set to approximately 30 mmHg using nitrogen for 24 h to create an elevated pressure model. Cells in the control group were not pretreated with Bromfenac. The culture media and cells were collected 24 h after placement of the cells in the pressure chamber.

### 2.2. Pressure-Induced Ischemia–Reperfusion (IR) Injury in the Optic Nerve and Retina of Rats

Male Sprague Dawley rats (7–8 weeks of age; weighing 250–300 g) were maintained on a 12:12 light/dark cycle under optimal temperature and humidity-controlled conditions. The rats were anesthetized using an intraperitoneal injection of 50 mg/kg ketamine with zolazepam (Zoletil; Virbac, Carros, France) and 15 mg/kg xylazine hydrochloride (Rompun; Bayer, Leverkusen, Germany). The anterior chamber of the test eye was cannulated with a 30-gauge infusion needle connected to a saline reservoir. The intraocular pressure (IOP) was increased to 120 mmHg for 60 min. Retinal ischemia was evident from the blanching of retinal arteries, as confirmed by fundus examination using an ophthalmoscope. When the needle was removed after 60 min, reperfusion of the retinal vasculature was confirmed using an ophthalmoscope. Postoperative antibiotic eyedrops were topically administered to prevent infection. The experimental eyes received a once-daily drop of 0.1% Bromfenac sodium hydrate, while the control eyes received a once-daily drop of phosphate-buffered saline (PBS).

All animals were cared for in accordance with the regulations of the Ethics Committee of the Catholic University and the Institutional Animal Care and Use Committee of the Catholic University of Korea. All experiments adhered to the Association for Research in Vision and Ophthalmology Statement for the Use of Animals in Ophthalmic and Vision Research. Additionally, we followed the National Institutes of Health Guide for the Care and Use of Laboratory Animals (NIH Publications, no. 80–23, revised 1996). A total of 104 animals were used, and careful management of the animals and procedures was ensured to minimize their number.

### 2.3. Enzyme-Linked Immunosorbent Assay (ELISA)

The cell culture supernatant obtained after centrifugation was utilized for subsequent studies. Protein concentrations of interleukin-1β (IL-1β), interleukin-6 (IL-6), and tumor necrosis factor-alpha (TNF-α) were measured using IL-1β, IL-6, and TNF ELISA Kits, respectively, following the manufacturer’s instructions. The analysis was performed using a microplate reader (Infinite M200 PRO, Tecan, Männedorf, Switzerland).

### 2.4. Western Blot Analysis

Cells or retinas were lysed in radioimmunoprecipitation assay (RIPA) buffer containing protease and phosphatase inhibitor cocktails. The RIPA buffer was composed of 50 mM Tris-HCl (pH 7.5), 150 mM NaCl, 1 mM EDTA, 0.1% SDS, 1% IGEPAL, and 0.5% sodium deoxycholate. Subsequently, the lysates were centrifuged at 10,000× *g* for 25 min at 4 °C, and the supernatants were assayed using a standard bicinchoninic acid assay (Pierce, Rockford, IL, USA). Retinal extracts underwent SDS–polyacrylamide gel electrophoresis and were then transferred onto a nitrocellulose membrane (Hybond-C, Amersham Pharmacia Biotech, Mannheim, Germany) and st`with Ponceau S (Sigma, St. Louis, MO, USA). Subsequently, the membranes were blocked for 45 min using 5% non-fat dry milk in Tris-buffered saline with Tween buffer (20 mM Tris-HCl, pH 7.6, 137 mM NaCl, and 0.1% Tween20). Blots were probed for 24 h with antibodies against ionized calcium-binding adapter-1 (Iba-1), glial fibrillary acidic protein (GFAP), COX-2, prostaglandin E2 receptor (PGE2-R), inducible nitric oxide synthase (iNOS), tumor necrosis factor-alpha (TNF-α), receptor-interacting protein (RIP) 1, RIP3, and β-actin. The blots were then incubated with horseradish peroxidase-conjugated goat secondary antibody for 1 h at room temperature. Protein bands were visualized using an enhanced chemiluminescence system (Amersham, Woburn, MA, USA) and X-ray film. The relative intensity of the bands was quantified using ImageMaster VDS (Pharmacia Biotech, City of Industry, CA, USA), and fold changes in protein levels were determined relative to β-actin.

### 2.5. Immunohistochemistry of Retinas

Both eyes were enucleated and immediately fixed in 4% paraformaldehyde at 4 °C for 10 min after sacrifice. Following removal of the anterior segment, the posterior segment was further fixed in 4% paraformaldehyde for 60 min. Scleral and retinal tissues embedded in OCT compound were then cryosectioned to a thickness of 6 µm. After multiple washes with phosphate-buffered saline (PBS), non-specific binding was blocked by incubating the sections with 10% normal donkey serum in PBS for 2 h at room temperature. Subsequently, the slides were incubated overnight at 4 °C with the following primary antibodies: anti-GFAP (Millipore, 1:200), anti-Iba-1(Millipore, 1:200), anti-iNOS (Sigma, 1:100), anti-PGE2-R (Sigma, 1:100), and anti-COX-2 (Sigma, 1:100). These antibodies were used to observe glial activation in the retinaGFAP (Millipore, 1:200), Iba-1(Millipore, 1:200), iNOS (Sigma, 1:100), PGE2-R (Sigma, 1:100), and COX-2 (Sigma, 1:100) were used to observe glial activation in the retina. The binding of primary antibodies was visualized using Alexa488- and Alexa546-conjugated secondary antibodies (Molecular Probes, Eugene, OR, USA). Following rinsing in 1× PBS, the slides were mounted with Fluoroshield mounting media containing DAPI (Vector Laboratories, Burlingame, CA, USA). Images of the stained tissues were captured using confocal laser scanning microscopy (Carl Zeiss, Jena, Germany).

Flat-mounted retinas were used for RGC-specific immunohistochemistry of mouse anti-brain-specific homeobox/POU domain protein 3a (anti-Brn3a; Millipore, Billerica, MA, USA, 1:200). After fixation in 4% paraformaldehyde, the intact retinas were extracted and washed with PBS. The immunostained retinas were then carefully flattened and mounted on microscopic slides using Fluoromount (Southern Biotech, Birmingham, AL, USA). Subsequently, the retinas were imaged using confocal laser scanning microscopy (Carl Zeiss). Each flat-mounted retina was divided into four equal quadrants. From the middle regions of each retinal quadrant, three fields measuring 200 × 250 µm^2^ were randomly sampled. Labeled ganglion cells were counted at 200× magnification in 12 regions of each retina.

### 2.6. Electroretinogram (ERG)

The rats were dark-adapted for 16 h. After anesthesia with Ketamine/Xylazine (100/10 mg/kg), the rats were placed in the LKC electroretinogram system (LKC Technologies Inc., Gaithersburg, MD, USA). The amplitudes and implicit times of the ERG waveforms were measured.

### 2.7. Statistical Analysis

All data are presented as means ± standard deviations (SDs). A two-sided Student’s *t*-test was employed to compare the control and experimental animals at each time point. A *p*-value of less than 0.05 was considered significant.

## 3. Results

### 3.1. Anti-Inflammatory Role of Bromfenac on Cultured Cells under Elevated Pressure

**Figure 1:**
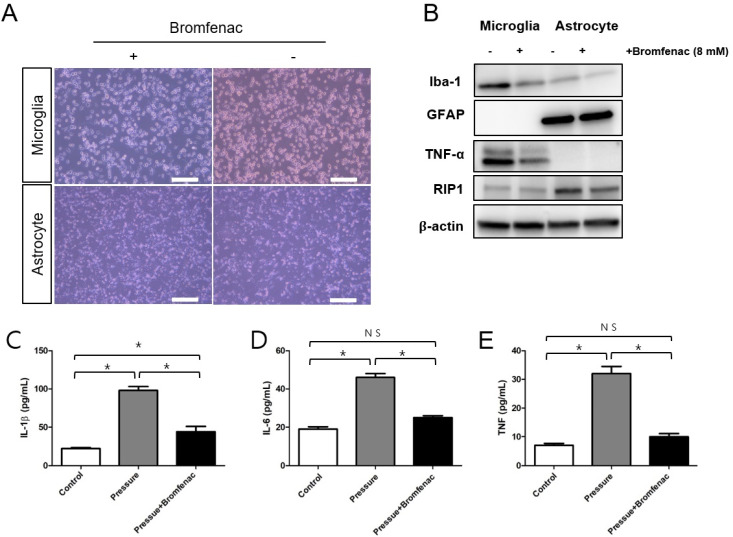
Cell culture in the pressure chamber with and without Bromfenac application. (**A**) Photograph of the cultures of microglia and astrocytes with and without Bromfenac administration under elevated pressure. For microglia, the scale bar represents 200 µm, while for astrocytes, the scale bar represents 500 µm. (**B**) Western blotting of proteins associated with glial cells under elevated pressure. With the administration of Bromfenac, Iba-1 and TNF-α expression decreased with microglia when RIP 1 expression decreased with astrocytes. For Western blotting analysis, n = 6 for control and n = 6 for Bromfenac administration; total n = 24. (**C**–**E**) Expression of IL-1β, IL-6, and TNF quantified using ELISA when cells were exposed to pressure with and without Bromfenac administration compared to the control. The expression of IL-1β, IL-6, and TNF increased significantly under elevated pressure and decreased significantly with Bromfenac administration. The bars represent the mean ± SD. Student’s *t*-test was used for the statistical evaluation. NS indicates not significant. * *p* < 0.05, for the specified comparisons.

To investigate the cellular responses of microglia and astrocytes under elevated pressure, with and without Bromfenac administration, the expression of inflammatory markers was investigated (Figure 1). Upon administration of Bromfenac, microglial proliferation was attenuated while astrocyte proliferation remained similar. Moreover, the use of Bromfenac resulted in a decrease in Iba-1, a marker of microglial cells, indicating reduced microglial activation. Additionally, TNF-α expression decreased in microglial cells following Bromfenac application. Following the administration of Bromfenac, GFAP expression, a marker of astrocytes, did not change. However, RIP 1 expression was decreased in the astrocytes after Bromfenac application (Figure 1B). The expression of IL-1β, IL-6, and TNF increased significantly in the culture media of both microglia and astrocytes under elevated pressure and all of them decreased significantly with Bromfenac administration (Figure 1C–E). These results suggest that glial cell activation or the trigger of neuroinflammation by elevated pressure was inhibited by Bromfenac application.

**Figure 2:**
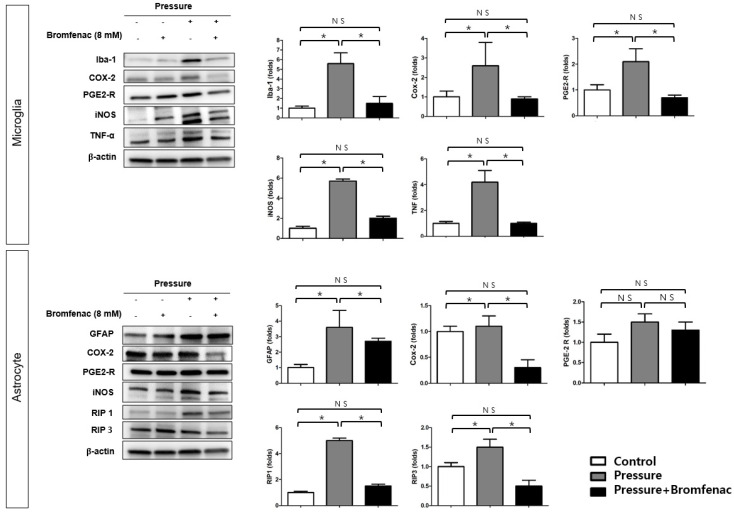
Western blot analysis of inflammatory marker expression in microglia and astrocytes in a pressure chamber with and without Bromfenac application. Western blot analysis was conducted to assess inflammatory marker expression in microglia and astrocytes under normal conditions, in a pressure chamber, with or without the administration of Bromfenac. The expression levels of Iba-1, COX-2, PGE2-R, iNOS, and TNF-α were significantly upregulated under elevated pressure conditions and significantly downregulated in the microglia when Bromfenac was administered. The expression levels of GFAP, COX-2, RIP 1, and RIP 3 were significantly upregulated under elevated pressure conditions and significantly downregulated in the astrocytes when Bromfenac was administered. The bars represent the mean ± SD. Student’s *t*-test was used for the statistical evaluation. NS indicates not significant. * *p* < 0.05, for the specified comparisons.

The levels of inflammatory markers were investigated using Western blotting in microglia and astrocytes under normal and pressure chamber conditions, with or without the administration of Bromfenac (Figure 2). Under pressure chamber conditions, the administration of Bromfenac led to significant downregulation of elevated levels of Iba-1, COX-2, PGE2-R, iNOS, and TNF-α in microglia. Similarly, in astrocytes under pressure chamber conditions, elevated levels of GFAP, COX-2, RIP 1, and RIP 3 were significantly downregulated following Bromfenac administration. These findings illustrate the individual downregulation of inflammatory markers in microglia and astrocytes separately, indicating that Bromfenac effectively suppresses neuroinflammation in both cell types. Especially, RIP 1 and RIP 3 were downregulated after Bromfenac administration in astrocytes, suggesting reduced necroptosis due to elevated pressure in this cell type.

### 3.2. Anti-Inflammatory Role of Bromfenac on IR Model

**Figure 3:**
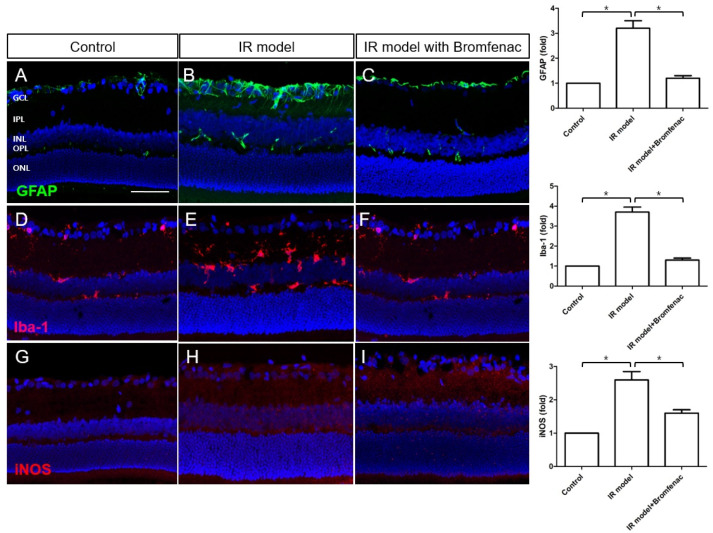
Immunohistochemical staining of GFAP, Iba-1, and iNOS in an ischemia–reperfusion (IR) injury model. (**A**,**D**,**G**) Control, (**B**,**E**,**H**) IR injury model, (**C**,**F**,**I**) IR injury model with Bromfenac. A significant increase in GFAP, Iba-1, and iNOS expression was noted in the IR injury model when all of them were downregulated by Bromfenac administration to the IR model. For immunohistochemical staining, the numbers of rats were n = 6 for the control, n = 6 for the IR model, and n = 6 for the IR model with Bromfenac administration, for a total n = 18. Scale bar = 100 µm. The bars represent the mean ± SD. Student’s *t*-test was used for the statistical evaluation. * *p* < 0.05, for the specified comparisons.

The results of the immunohistochemical staining provide evidence for the anti-inflammatory effect of Bromfenac in a glaucoma animal model subjected to IR injury. In the IR model, there was an observed increase in the expression of GFAP, Iba-1, and iNOS (Figure 3B,E,H), indicating glial activation and neuroinflammation, which are characteristic features of this injury model. However, upon Bromfenac treatment, significant decreases in the expression of GFAP, Iba-1, and iNOS were noted (Figure 3C,F,I).

**Figure 4:**
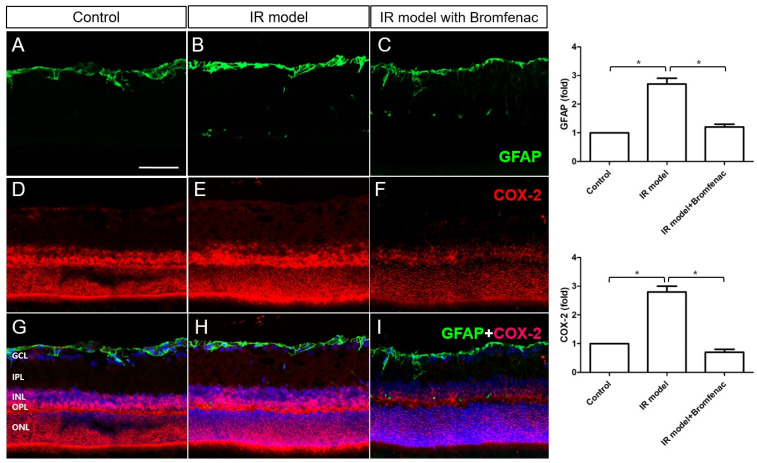
Immunohistochemical staining for GFAP and COX-2 in an ischemia–reperfusion (IR) injury model. (**A**,**D**,**G**) Control, (**B**,**E**,**H**) IR injury model, (**C**,**F**,**I**) IR injury model with Bromfenac. A significant increase in GFAP and COX-2 expression was noted in the IR injury model when both of them were downregulated by Bromfenac administration. For immunohistochemical staining, the numbers of rats were n = 6 for the control, n = 6 for the IR model, and n = 6 for the IR model with Bromfenac administration, for a total n = 18. Scale bar = 100 µm. The bars represent the mean ± SD. Student’s *t*-test was used for the statistical evaluation. * *p* < 0.05, for the specified comparisons.

**Figure 5:**
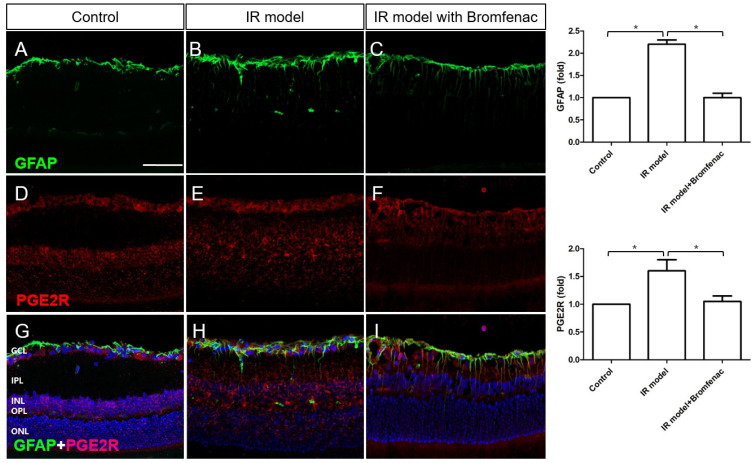
Immunohistochemical staining for GFAP and PGE2-R in an ischemia–reperfusion (IR) injury model. (**A**,**D**,**G**) Control, (**B**,**E**,**H**) IR injury model, (**C**,**F**,**I**) IR injury model with Bromfenac. A significant increase in GFAP and PGE2-R expression was noted in the IR injury model when both of them were downregulated by Bromfenac administration. For immunohistochemical staining, the numbers of rats were n = 6 for the control, n = 6 for the IR model, and n = 6 for the IR model with Bromfenac administration, for a total n = 18. Scale bar = 100 µm. The bars represent the mean ± SD. Student’s *t*-test was used for the statistical evaluation. * *p* < 0.05, for the specified comparisons.

We used immunohistochemical staining to examine the levels of COX-2, PGE-2R, and GFAP (Figure 4 and Figure 5). Our findings revealed an elevation in COX-2 and PGE-2R expression within astrocytes in the IR model, whereas their expression declined with Bromfenac treatment. Since Bromfenac works by inhibiting the enzyme COX, thereby reducing prostaglandin E2 (PGE_2_) production, this suggests that Bromfenac suppresses neuroinflammation in the IR model. Consequently, the downregulation of PGE-2R further supports this notion, indicating a cascade effect of Bromfenac’s mechanism of action in inhibiting COX and subsequently reducing PGE_2_ levels.

### 3.3. Neuroprotective Effect of Bromfenac on RGCs

**Figure 6:**
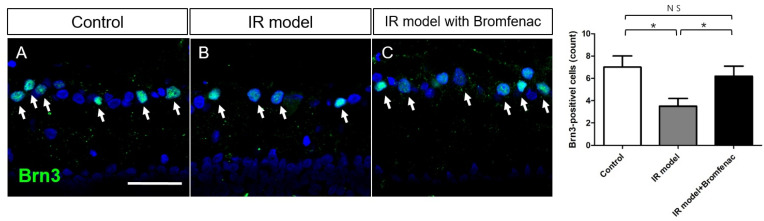
Brn3a immunohistochemical staining of retinal ganglion cells (RGCs) on flat-mounted retinas. (**A**) Control, (**B**) IR injury model, (**C**) IR injury model with Bromfenac. There was a significant RGC loss in the IR injury model when an IR model with Bromfenac administered exhibited a preserved number of RGCs. White arrows indicate RGCs. Scale bar = 50 µm. The bars represent the mean ± SD. Student’s *t*-test was used for the statistical evaluation. NS indicates not significant. * *p* < 0.05, for the specified comparisons.

To determine the neuroprotective effect of Bromfenac on RGCs, Brn3 immunohistochemical staining was performed on flat-mounted retinas (Figure 6). A decrease in the number of RGCs was observed following IR injury compared to that in the normal retina. With Bromfenac treatment, however, a significant increase in the number of RGCs was observed, which was comparable to that of the control. These findings suggest that glial activation and pro-inflammatory status following IR injury were successfully inhibited by the anti-inflammatory effect of Bromfenac, leading to neuroprotection and resulting in increased RGC survival.

### 3.4. Neuroprotective Effect of Bromfenac on Electrophysiology

**Figure 7:**
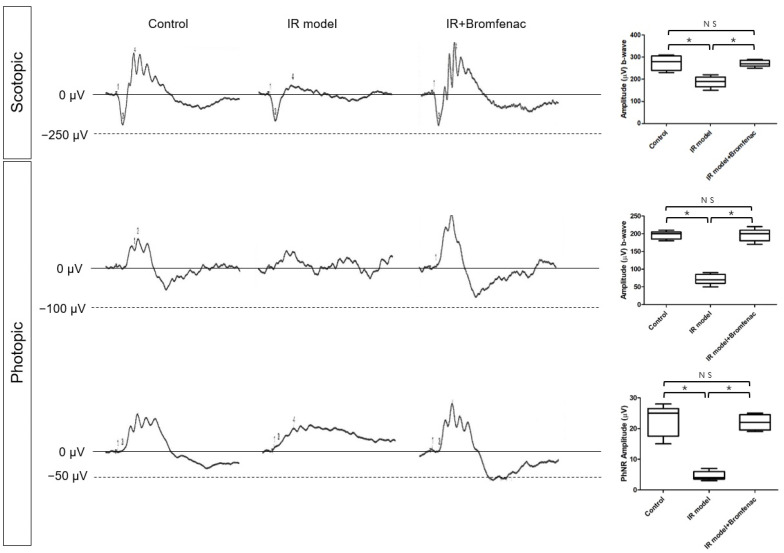
Scotopic and photopic electroretinograms (ERGs) of the control, ischemia–reperfusion (IR) injury model, and IR model with Bromfenac. The b-wave amplitude was significantly decreased in the scotopic and photopic ERG of the IR model compared to that of the control. When Bromfenac was administered to the IR model, the b-wave amplitude recovered on both the scotopic and photopic ERGs. The PhNR amplitude was significantly decreased in the IR model and recovered when Bromfenac was administered to the IR model. The bars represent the mean ± SD. Student’s *t*-test was used for the statistical evaluation. NS indicates not significant. * *p* < 0.05, for the specified comparisons.

To evaluate the neuroprotective effects of Bromfenac in vivo, we conducted ERG assessments on three groups of animals: control, IR model, and IR model treated with Bromfenac (Figure 7). The b-wave amplitude of scotopic ERG and photopic ERG parameters, including b-wave and photopic negative response (PhNR) amplitude, were significantly decreased in the IR model group compared to the control group. However, upon treatment with Bromfenac, all these measures exhibited a significant increase compared to the IR model group, reaching levels comparable to those of the control group. As PhNR represents the electrical activity of the RGC layer [18], these findings were consistent with immunohistochemical staining, demonstrating enhanced RGC survival with Bromfenac administration. By preserving retinal function on the ERG, the neuroprotective effect of Bromfenac was established in vivo, not only at the cellular or histological level but also at the functional level of the animal.

## 4. Discussion

This study investigated the impact of Bromfenac on diminishing neuroinflammation in an IR glaucoma model. We explored its effects at the cellular level, specifically on microglia and astrocytes. Bromfenac effectively suppressed the expression of inflammatory cytokines, which were elevated in a simulated pressure chamber environment, indicative of neuroinflammation. Additionally, necroptosis markers were downregulated using Bromfenac in astrocytes. In the IR model, Bromfenac demonstrated the attenuation of glial activation and neuroinflammation. This was achieved through its ability to suppress iNOS upregulation and inhibit COX-2 to reduce the expression of PGE2, subsequently leading to the downregulation of PGE2-R. The successful inhibition of neuroinflammation was correlated with the enhanced survival of RGCs and the preservation of retinal function, as confirmed by ERG.

The involvement of nitric oxide (NO) in the pathophysiology of glaucoma is intricate, exhibiting a dual role contingent upon physiological and pathological contexts [19,20]. Physiologically, a low dose of NO demonstrates neuroprotective properties, and accumulating evidence underscores its efficacy in reducing IOP by targeting conventional outflow tissues [21,22]. Notably, the approval of latanoprostene bunod by the United States Food and Drug Administration (FDA) in 2017, a NO-donating prostaglandin F receptor agonist, surpassing the efficacy of latanoprost, confirms the dual mode of action involving NO donation and FP receptor activation in IOP reduction [23,24]. Conversely, during pathological conditions characterized by stimuli such as inflammation or ischemia, iNOS activation leads to the generation of excessive NO, resulting in the formation of peroxynitrite and other reactive nitrogen species alongside superoxide free radicals [25,26]. In previous studies, iNOS upregulation has been observed in activated astrocytes within the glaucomatous ONH [27], as well as in instances of elevated IOP following the cauterization of episcleral veins [28] and in the retina subsequent to ischemic events [29]. The inhibition of iNOS prevented the loss of RGCs in a vein cauterization rat model [30]. Our results are consistent with those of these studies, with the upregulation of iNOS in the IR model. Moreover, we demonstrated a reduction in iNOS upregulation upon the administration of Bromfenac, suggesting a reduction in neuroinflammation. Given the paradoxical roles of NO as both an IOP-lowering agent and a mediator of neuroinflammation, comprehensive in vivo studies are needed to elucidate the precise levels of NO mediating these disparate effects.

Prostaglandins, which are small pro-inflammatory molecules synthesized by COX enzymes, play crucial roles in various physiological processes, including inflammation and pain modulation [31]. Within the ophthalmology domain, prostaglandin analogs serve as essential medications for lowering IOP in glaucoma management. They achieve this by increasing the uveoscleral outflow of the aqueous humor. While prostaglandin F2 is conventionally utilized for treating ocular hypertension, the recent approval by the FDA in 2022 of omidenepag isopropyl, a selective agonist for prostaglandin E2 receptor 2 (EP2), one of the four PGE2-Rs, marks a significant advancement in glaucoma therapy [32]. Notably, our study has demonstrated a decrease in PGE2-R expression in models of IR injury upon the administration of Bromfenac, leading to the enhanced survival of RGCs. However, there may be a misconception regarding the specific targeting of one of the PGE2-Rs by the newly approved glaucoma medication. PGE2, a versatile prostanoid, exerts diverse effects through multiple receptor subtypes with varying affinities, often displaying functionally opposing directions within the same cell or organ [33]. Elevated PGE2 levels are implicated in various neurological disorders, contributing to neuronal death via direct or indirect neurotoxicity, glutamate receptor-mediated excitotoxicity, and innate immune activation [34]. Thus, elucidating the intricate signaling cascade involving prostaglandins and Bromfenac within the context of neuroinflammation is essential for a comprehensive understanding of their therapeutic mechanisms.

The conventional understanding of RGC death in glaucoma involves apoptotic mechanisms. However, in our previous study [12], RGC death in glaucoma cases with unstable hemodynamic characteristics involved necroptosis mediated by TNF-α and RIP 3. Necroptosis is one of the best-characterized forms of regulated necrosis [35]. Other than apoptosis, necroptosis directly triggers inflammation by a massive release of damage-associated molecular patterns (DAMPs) from the disrupting barriers [36]. Following Bromfenac administration under elevated pressure conditions, key molecules in the necroptosis cascade, RIP 1 and RIP 3, were observed to be downregulated in astrocytes. From this perspective, Bromfenac shows potential for neuroprotection by reducing necroptosis.

This study demonstrates a significant strength in its utilization of a widely available commercial eyedrop, Bromfenac, thereby enhancing its practical applicability. Topical administration of medications remains the preferred approach for managing ocular inflammation due to its ability to achieve higher concentrations within the eye while minimizing systemic side effects. Although corticosteroids are commonly used for topical therapy of ocular inflammation, their propensity to elevate IOP poses a significant challenge, particularly in patients with glaucoma [37,38]. NSAIDs have emerged as safe alternatives for the topical management of ocular inflammation. They find extensive application in various clinical scenarios, including postoperative inflammation, seasonal allergic conjunctivitis, prevention and treatment of cystoid macular edema, and pain control after photorefractive keratectomy [39,40]. Adverse events frequently associated with Bromfenac ophthalmic solution, including abnormal sensations in the eye, conjunctival hyperemia, eye irritation, pain, eye pruritus, eye redness, headache, and iritis, were not linked to vision-threatening or serious problems. These occurrences were observed in 2% to 7% of patients [41].

In the context of glaucoma, where the primary focus is on reducing IOP through eyedrops, patients frequently encounter discomfort associated with these medications. Bromfenac stands out as a relatively safe option for managing ocular inflammation. Furthermore, our study indicates its potential to concurrently mitigate neuroinflammation, thereby exerting a neuroprotective effect that could enhance RGC survival. Further research is essential to comprehensively understand Bromfenac’s neuroprotective attributes in human patients with glaucoma.

## 5. Conclusions

Bromfenac successfully reduced neuroinflammation at the cellular level in microglia and astrocytes under elevated pressure, as demonstrated in an IR glaucoma animal model. This led to the improved survival of RGCs, indicating Bromfenac’s neuroprotective potential. Furthermore, an in vivo assessment revealed the preservation of ERG retinal function, affirming its efficacy. These findings support the potential therapeutic role of Bromfenac in mitigating the neuroinflammation associated with glaucoma, which could have implications for the development of novel treatment strategies targeting inflammation in this disease.

## Data Availability

Data may be provided upon reasonable request.
